# The Role of Cdc42 in the Insulin and Leptin Pathways Contributing to the Development of Age-Related Obesity

**DOI:** 10.3390/nu15234964

**Published:** 2023-11-29

**Authors:** Bauyrzhan Umbayev, Timur Saliev, Yuliya Safarova (Yantsen), Aislu Yermekova, Farkhad Olzhayev, Denis Bulanin, Andrey Tsoy, Sholpan Askarova

**Affiliations:** 1 National Laboratory Astana, Nazarbayev University, Astana 010000, Kazakhstan; yantsen@nu.edu.kz (Y.S.); aislu.yermekova@nu.edu.kz (A.Y.); folzhayev@nu.edu.kz (F.O.); andrey.tsoy@nu.edu.kz (A.T.); shaskarova@nu.edu.kz (S.A.); 2 S.D. Asfendiyarov Kazakh National Medical University, Almaty 050012, Kazakhstan; tim.saliev@gmail.com; 3 Department of Biomedical Sciences, School of Medicine, Nazarbayev University, Astana 010000, Kazakhstan; dbulanin@nu.edu.kz

**Keywords:** Cdc42, insulin–leptin axis, age-related obesity, aging, leptin resistance, insulin resistance

## Abstract

Age-related obesity significantly increases the risk of chronic diseases such as type 2 diabetes, cardiovascular diseases, hypertension, and certain cancers. The insulin–leptin axis is crucial in understanding metabolic disturbances associated with age-related obesity. Rho GTPase Cdc42 is a member of the Rho family of GTPases that participates in many cellular processes including, but not limited to, regulation of actin cytoskeleton, vesicle trafficking, cell polarity, morphology, proliferation, motility, and migration. Cdc42 functions as an integral part of regulating insulin secretion and aging. Some novel roles for Cdc42 have also been recently identified in maintaining glucose metabolism, where Cdc42 is involved in controlling blood glucose levels in metabolically active tissues, including skeletal muscle, adipose tissue, pancreas, etc., which puts this protein in line with other critical regulators of glucose metabolism. Importantly, Cdc42 plays a vital role in cellular processes associated with the insulin and leptin signaling pathways, which are integral elements involved in obesity development if misregulated. Additionally, a change in Cdc42 activity may affect senescence, thus contributing to disorders associated with aging. This review explores the complex relationships among age-associated obesity, the insulin–leptin axis, and the Cdc42 signaling pathway. This article sheds light on the vast molecular web that supports metabolic dysregulation in aging people. In addition, it also discusses the potential therapeutic implications of the Cdc42 pathway to mitigate obesity since some new data suggest that inhibition of Cdc42 using antidiabetic drugs or antioxidants may promote weight loss in overweight or obese patients.

## 1. Introduction

Obesity is an excessive fat deposition in the body, and it can be considered an independent multifactorial disease or a syndrome accompanying other conditions. In 1997, WHO proposed the most common classification of obesity based on body mass index (BMI (kg/m^2^)) [1]. Such a definition describes obesity as having a BMI more than or equal to 30, whereas overweight is defined as having a BMI larger than or equal to 25. The global prevalence of obesity and the burden of its comorbidities is increasing [2,3]. In 2016, according to WHO, over 650 million of the World’s adult population (11% of men and 15% of women) were obese [1].

Although obesity is a disorder that affects people of all ages, many epidemiological studies have found an increased risk of obesity from puberty until late middle age and a decrease after that [4,5,6,7,8]. Moreover, it has been established that obesity accelerates the aging process [9]. Multiple studies have demonstrated similar physiological mechanisms underlying obesity and aging [4,5,6,7,10,11,12,13,14,15,16,17,18,19,20,21]. The results of recent studies suggest that aging is the most substantial risk factor for developing metabolic syndrome and obesity [9,22]. Nevertheless, the influence of aging processes on the development of obesity is not well understood.

Age-related obesity is a complex process that depends on many factors, among which hormones such as leptin and insulin play a pivotal role [23]. Leptin regulates appetite, food intake, and energy balance by activating specific neuronal signals in the hypothalamus [24,25]. According to the early hypothesis, obesity occurs due to hypothalamic dysregulation of food intake and energy homeostasis caused by dysregulation of the adipokine leptin and decreased tissue sensitivity to its action, and hyperleptinemia and leptin resistance develop with increasing age [10,26,27,28,29,30,31,32]. Thus, leptin resistance may play a crucial role in age-associated weight gain and obesity, although underlying mechanisms are poorly understood.

In turn, hyperinsulinemia and insulin resistance are significant hallmarks of aging and obesity [33]. The relationships between insulin and its metabolic partner, leptin, play an essential role in energy homeostasis [34]. It has been shown that chronic insulin exposure can induce leptin secretion and production [35,36,37,38,39] and hyperinsulinemia causes leptin resistance in hippocampal neurons [34,40,41,42]. Vice versa, leptin can influence insulin and insulin resistance [43,44]. Although some researchers have not found an association between insulin and leptin levels [45,46], most in vitro, in vivo, and clinical studies indicate that there is a close relationship between these two hormones, and the role of leptin in age-related obesity is insulin-dependent [34,35,36,37,38,39,40,41,42]. Yet the interactions between these key regulators of energy metabolism are complex and poorly understood [47]. In this regard, the signaling pathways common to insulin and leptin, particularly the primary molecular players involved in the crosstalk between insulin, leptin, and other factors, require special attention.

Recent findings have pointed to the Rho GTPase Cdc42 as an integral part of regulating insulin secretion [48] and aging processes [49]. Cdc42 is a member of the Rho family of GTPases that regulates actin cytoskeleton, vesicle trafficking, cell polarity, morphology, proliferation, motility, and migration [50]. Upon activation, Cdc42 changes from an inactive GDP-bound form to an active GTP-bound state and back again, and these transformations are controlled by various proteins and regulatory factors [51]. The active form of Cdc42-GTP interacts with multiple effector proteins (P21-activated kinases, WASP and N-WASP, IQGAPs, MRCK, NADPH, Par proteins, etc.) and participates in signaling pathways that regulate growth, survival, differentiation, and many other aspects of cell biology [51]. Therefore, disturbances in the regulation of Cdc42 are implicated in many diseases, including cancer and neurodegenerative diseases [49,52].

Although the role of Cdc42 in glucose metabolism and interactions with insulin are relatively well understood, the results of the studies on Cdc42 and leptin interactions represent scattered data. Therefore, the review aims to summarize current knowledge on the role of Cdc42 in the crosstalk between the insulin and leptin pathways contributing to the development of age-related obesity. In addition, this article discusses the potential therapeutic implications of the Cdc42 pathway to mitigate obesity.

## 2. Age-Related Obesity and Insulin Imbalance

The relationship of human weight with age has two distinct patterns: an increase in the prevalence of obesity in middle age [53,54], and anorexia, weight loss, sarcopenia, and senile cachexia in the elderly after age 75 [55]. In general, people aged 75 years and older have a lower prevalence of obesity compared with 65–74 years old adults [56]. The results of multiple cross-sectional national surveys and a national longitudinal study of Australian women, which started in 1996 and involved more than 57,000 women in four age cohorts, have demonstrated that age-associated weight gain lasts from adolescence until late middle age [11]. Similar effects have been observed in nonhuman primates [12,13], dogs [14], cats [15], rats [16], and mice [17,18,19,20,21]. An Australian longitudinal study has also demonstrated that being overweight in middle age is strongly associated with a higher incidence of chronic diseases [57]. This finding agrees with a recent prospective cohort study in the US showing that overweighed middle-aged people have a higher burden of morbidity yet unaffected overall life expectancy. In comparison, in obese people, a higher burden of morbidity is accompanied by a shorter overall life expectancy [58]. In addition, modeling the trajectory of BMI over 28 years in women revealed an increased risk of dementia in obese women in their 50s but not in their 60s or 70s [59].

One of the reasons for the tendency of people in middle age to become overweight or obese is an age-related increase in total fat mass [60,61,62,63], which may also occur independently of gaining weight [64,65,66,67]. Total human fat mass consists mainly of two major compartments of white adipose tissue: subcutaneous adipose tissue (SAT) and visceral adipose tissue (VAT). Significant anatomical, cellular, molecular, physiologic, clinical, and prognostic differences exist in these adipose tissues in the body [68]. Numerous studies have demonstrated that VAT is a pro-inflammatory tissue with a higher number of inflammatory macrophages than SAT. It is associated with metaflammation (systemic and local inflammation), which can be quantified in organs [60,61,62,68]. In turn, with age, fat tends to accumulate predominantly in the abdominal and visceral areas and penetrate into muscles and bones [63,69], with noticeable gender differences in the dynamics of subcutaneous and visceral adipose tissue distribution. For instance, early studies have indicated that men reach their maximum subcutaneous fat volume at 40–50 years, while women up to 70 years [70]. Furthermore, in women, there is a sharp, almost four-fold increase in the amount of visceral fat between the ages of 25 and 65 years [71], while in men the mass of visceral adipose tissue increases only two-fold over the same period [72]. Although fat accumulation in adulthood is associated with many factors including decreased physical activity, high-calorie diet, stress, sleep patterns, etc., the crucial contributor to age-related obesity is a shift in hormonal balance and consequent reduction in the metabolic rate [73,74,75]. Extensive research and clinical studies have shown that age-related complex changes in the endocrine system affect the secretory pattern of hormones, tissue sensitivity to hormones, and imbalance of hormone levels [76,77,78].

The expansion of total fat mass depends on adipocyte proliferation (hyperplasia) and an enlargement of adipocyte size (hypertrophy) [79]. The results of several studies have found that there is a balance between hyperplasia and hypertrophy in different adipose tissue depots [64,65]. In general, hypertrophy precedes hyperplasia, and when adipocytes reach the lipid accumulation limit, adipocyte proliferation and/or differentiation are triggered [66,67]. An imbalance between hyperplasia and hypertrophy may exacerbate the metabolic outcome of obesity [80,81,82]. Insulin is one of the main endocrine hormones regulating energy and lipid metabolism, and it is actively involved in stimulating both hypertrophy and hyperplasia of adipocytes [83,84,85]. Exposure to insulin leads to the elevation of the absorption of glucose, as well as the storage of free fatty acids and triglycerides in adipocytes [86]. In addition, insulin suppresses lipolysis and promotes de novo synthesis of fatty acids in adipose tissue cells [87]. Insulin is vital to the late stages of adipogenesis because it enhances gene expression of various fat-specific transcription factors and is required to achieve a fully functional adipocyte phenotype [88,89,90].

Gender differences in age-related obesity between men and women may also originate from the differences in insulin secretion and tissue sensitivity to insulin [91,92]. Glucose-stimulated insulin secretion patterns may vary, with women exhibiting a higher level of secretion [93]. Studies show that women are generally more insulin-sensitive than men, while males have more insulin and high-affinity receptors [94,95,96]. As individuals age, both genders experience a decrease in peripheral insulin sensitivity [96,97]. Sex hormones positively affect insulin, so while testosterone sensitizes tissues to insulin [98], estrogens, in addition to increasing tissue sensitivity to insulin, also stimulate the synthesis and release of insulin by the pancreas [99]. Insulin resistance impacts both men and women, with a higher prevalence observed in men. However, following menopause, the incidence in women rises and becomes comparable to that in men. Changes occurring postmenopausal may heighten the risk of insulin resistance and the development of type 2 diabetes [100].

Hyperinsulinemia, also known as dysregulated insulin production and/or clearance, is a prevalent feature of obesity and metabolic diseases that results in persistently increased insulin levels without hypoglycemia [101,102]. Hyperinsulinemia is multifactorial and related to a number of causes, including genetic predisposition, lifestyle, diet, and environment [101]. Several epidemiological and laboratory studies have shown a complex relationship between age and insulin secretion and clearance: while increased insulin secretion and hyperinsulinemia are more prominent in late middle age, decreased insulin secretion is observed in advanced old age [103,104,105,106,107,108]. On the other hand, the data about the effect of aging on insulin clearance are contradictory: some studies find no difference [105,109], while others report a decreased insulin clearance in aged rodents and humans [110,111,112]. Kurauti et al. hypothesized that insulin clearance is not impaired at younger ages due to compensatory activation of insulin-degrading enzyme (IDE) expression. However, at later ages, this compensatory mechanism breaks down, leading to decreased insulin clearance and increased hyperinsulinemia [103,105].

Aging and obesity cause immune dysregulation in adipose tissue, leading to chronic inflammation with increased infiltration and activation of innate and adaptive immune cells [113,114,115,116]. The number of macrophages, the primary adipose tissue immune cell population, increases with fat mass accumulation and aging, potentially accounting for up to 40% of all adipose tissue cells [117,118,119]. Adipose tissue chronic inflammation is triggered by the secretion of several inflammatory factors and an increase in pro-inflammatory macrophages in the adipose tissue associated with obesity [120,121]. Although the link between insulin resistance and inflammation remains unclear, data demonstrate that inflammation can lead to the development of insulin resistance [122]. Furthermore, it has been shown that proinflammatory cytokines can also stimulate insulin production [123]. In turn, hyperinsulinemia and insulin resistance may promote myelinogenesis and common myeloid progenitor (CMP) granulocyte/monocyte progenitor (GMP) proliferation through the FoxO signaling pathway [124]. Yet it has recently been shown that insulin resistance triggered by obesity emerges before the accumulation of macrophages and the onset of inflammation in adipose tissue [125].

## 3. Age-Related Obesity and Leptin Resistance

The age-related increase in fat mass and hyperinsulinemia triggers an imbalance of leptin-dependent regulation of adipose tissue homeostasis [126]. Leptin is a member of a family of long-chain class-I helical cytokines that includes the IL-6, G-CSF, erythropoietin, thrombopoietin, growth hormone, and prolactin [127]. Leptin is found in all mammals and is characterized by a highly preserved primary amino acid sequence and its functions [128]. The human LEP gene is located on chromosome 7 and encodes a 167 amino acid peptide with a molecular weight of 16 kD [129,130]. Leptin is produced mainly by adipocytes and is considered a crucial adipostatic factor. Under normal physiologic conditions, high leptin levels decrease adipose tissue mass by activating hypothalamic control of energy expenditure, thermogenesis, and calorie intake [25,131,132]. Therefore, serum levels of leptin are proportional to the amount of energy stored in adipose tissue and fluctuate with significant changes in calorie intake [133,134]. However, in obesity and aging, leptin malfunction is associated with decreased sensitivity of tissues to leptin [25].

Leptins bind to the obesity receptor (Ob-R). There are six isoforms of Ob-R divided into three classes (long, short, and secretory isoforms), which are the products of alternative RNA splicing of the db gene. A long, fully active isoform of Ob-Rb is expressed mainly in the hypothalamus, where it takes part in energy homeostasis and in the regulation of secretory organ activity. Ob-Rb is also present in all types of immune cells and is involved in innate and adaptive immunity. Short leptin isoforms co-called Ob-Ra, Ob-Rc, and Ob-Rd are able to bind JAK kinases and activate some signal transduction cascades. A soluble isoform (Ob-Re) can regulate serum leptin concentration and serve as a carrier protein delivering the hormone to its membrane receptors and is able to transduce the signal into the cell [135]. Gancarz et al. found that aging decreases the soluble form of Ob-Re and Ob-Ra in human monocytes, while the long form of the leptin receptor Ob-Rb remains unchanged [136].

Over the past 20 years, the classic hypothalamic leptin–melanocortin model of leptin’s anorexigenic effects has been proposed [137,138]. Within this model, leptin is thought to bind to its receptor, the so-called long form of the leptin receptor (Ob-Rb), which is expressed by two antagonistic populations of neurons in the arcuate nucleus of the hypothalamus (ARC): proopiomelanocortin (POMC)-containing neurons and neuropeptide Y (NPY)/agouti-related peptide (AgRP) (AGRP/NPY) neurons [139,140]. Leptin binding to Ob-Rb leads to activation of POMC neurons, which produce anorexigenic molecules such as αMSH (α-melanocyte-stimulating hormone) and deactivation of AGRP/NPY neurons. This results in decreased food intake and energy expenditure. At low leptin levels, there is deactivation of POMC neurons and activation of AGRP/NPY neurons, which produce AGRP/NPY orexigenic peptides, resulting in increased appetite. However, recent studies demonstrated that this model is insufficient for understanding leptin regulation of energy homeostasis [137,141]. A line of evidence has shown that leptin’s effects on eating behavior are not due to direct effects on POMC neurons [137,142,143]. While the direct action of leptin on POMC neurons is more likely related to the maintenance of normal glucose homeostasis [142,144], it is hypothesized that the specific effects of leptin are mediated by multiple populations of leptin-sensitive non-POMC neurons [137,145,146,147,148]. Thus, the current understanding of leptin’s regulation of hypothalamic neuronal circuits that control feeding and energy expenditure is incomplete and needs to be revised.

After discovering leptin in 1994, it was hypothesized that increased leptin concentrations should have led to decreased appetite and increased energy expenditure in obesity [25]. However, treatment with leptin in obesity did not produce the expected effects. To explain this paradox, the concept of leptin resistance has been proposed by analogy with insulin resistance [30,133]. Leptin resistance is described as a diminished sensitivity or lack of response to endogenous or exogenous leptin. It is characterized by high circulating leptin concentrations (hyperleptinemia) and a decreased tissue sensitivity to leptin [28,30,149]. Traditionally, two main types of leptin resistance are distinguished: reduced tissue sensitivity to leptin in the brain (central leptin resistance) and in the peripheral tissues (peripheral leptin resistance) [150]. The development of leptin resistance requires a combination of high endogenous leptin levels in the blood and a high-fat diet; separately, these factors cannot lead to the development of leptin resistance [151].

A number of studies indicate that aging is a risk factor for the development of leptin resistance and obesity. However, the causative relations between leptin resistance and age-related obesity are disputable [28,29,152,153,154,155]. Some authors believe leptin resistance develops in response to obesity, while some findings suggest that obesity develops in response to aging-associated leptin resistance. For instance, a recent study found that both male and female Wistar rats showed an age-related increase in plasma leptin concentrations [156]. Transgenic mice with chronically elevated leptin levels exhibited a lean phenotype at a young age, whereas at 33–37 weeks, there was an increase in body weight, significant accumulation of fat mass, and lipid deposition in adipocytes [157]. Studies in male F-344 × BN rats have shown that leptin gene expression in inguinal white adipose tissue and circulating leptin levels increase with age without increasing adiposity [16]. In contrast, in C57BL/6J mice, both serum leptin concentrations and leptin gene expression levels decreased with age, while body weight remained stable [17]. Similarly, Ma et al. showed that basal leptin levels in the blood of F-344 × BN rats were two-fold higher in 4-month-old F344 × BN rats compared with 21-month-old rats. At the same time, an exogenous increase in plasma leptin levels resulted in a 50% suppression of leptin gene expression in younger animals. Still, it did not inhibit leptin gene expression in aging rats [27]. Yet these conflicting results may be due to differences in study design based on normalization of leptin levels [158]. Moreover, several lines of evidence suggest that age-related changes in leptin levels in humans differ significantly from their rodent counterparts [29,158].

It is worth mentioning that men and women differ in the leptin levels they synthesize and secrete and in their responses to endogenous and exogenous leptin [159,160]. Several studies indicate that women have higher plasma leptin levels than men, regardless of the variation in total body fat mass [159,161]. The predominance of subcutaneous adipose tissue in women, which is distinguished not only by large mass but also by greater expression and secretion of leptin compared with men, is thought to be the source of sexual dimorphism in leptin levels [159,161,162]. Estrogens enhance leptin sensitivity [163], while androgens induce leptin resistance and dysfunction [164,165,166]. It has been found that estradiol and estradiol receptor levels drop with age, especially during menopause [167]. It is hypothesized that the age-related decrease in estradiol receptor expression reduces the effect of this hormone on leptin [168]. Testosterone is also likely to play a crucial role in sexual dimorphism, reducing the growth of subcutaneous fat tissue, leptin secretion, and gene expression [164,165,166]. Males and females respond differently to leptin, with female brains being more sensitive to it [169]. Estradiol may enhance hypothalamic expression of the long form of the leptin receptor, potentially modulating central leptin sensitivity in female gonadal hormones [170]. When exposed to a high-fat diet, males develop leptin resistance earlier than females [171,172,173].

The causes of a decrease in tissue leptin sensitivity can generally be classified into two mechanisms: the first is linked to impaired transport of leptin to its receptors, while the second includes mechanisms leading to a decrease in signal transmission from the receptor to the downstream effectors [149,174]. Leptin is subsequently released into the bloodstream, where it can exist in a free active form and an inactive form bound to leptin-binding proteins Ob-Re [175,176,177,178]. The ratio of a free form of leptin to Ob-Re is an important indicator of leptin bioavailability called the free leptin index (FLI = leptin/Ob-Re) [179]. The concentration of human OB-Re in serum is proportional to the amount of membrane-bound leptin receptor [180]. It is formed by proteolytic cleavage of the membrane-bound leptin receptor by metalloproteases, mainly in the liver [181].

Obesity leads to a decrease in Ob-Re levels and an increase in FLI [178,182,183,184]. In turn, decreased blood Ob-Re levels are associated with leptin resistance linked to obesity and diabetes [185]. Lean individuals have more bound leptin in their blood, and weight loss is associated with increased levels of Ob-Re in the blood of both humans and rodents. It is hypothesized that decreased Ob-Re levels in obese and diabetic patients lead to increased leptin clearance and may enhance leptin-induced leptin resistance [181]. Some data suggest that an imbalance between the free form of leptin and Ob-Re may be one of the most important mechanisms for the development of leptin resistance [47,186]. Several studies have also shown that FLI seems to change with age [136,187,188]. For instance, a 2015 study found that the median FLI was substantially greater in healthy elderly, non-obese males (aged 64.7 ± 3.1 years) than in young males (aged 26.8 ± 3.6 years) [136]. Leptin-binding activity levels were shown to be low at birth, high in the years before puberty, reduced through puberty, and then remained steady throughout adulthood [188].

A likely reason for the drop in Ob-Re levels in the blood during obesity and aging may be a combination of chronic hyperleptinemia and endoplasmic reticulum stress (ER stress) in hepatocytes [189]. Exposure to leptin can lead to temporary ligand-induced suppression of the receptor [190]. Under normal conditions, the membrane pool of the leptin receptor is replenished. Still, with obesity and aging, misfolded proteins accumulate in the hepatocytes’ endoplasmic reticulum, the so-called ER stress, which slows the return of leptin receptors to the plasma membrane [189]. A recent study suggests that activation of the endocannabinoid CB1R system in the liver, which induces ER stress and suppresses CHOP protein, may reduce Ob-Re expression in hepatocytes [186]. The CB1R system, linked to metabolic syndrome, including leptin and insulin resistance, is triggered by both central and peripheral stimulations during obesity [191,192]. Aging leads to increased metabolic signatures of chronic ER stress, and in the liver, in particular, decreased ER stress with age may contribute to metabolic diseases [193].

In turn, multiple mechanisms of energy homeostasis regulation are triggered by binding leptin to its membrane-bound receptor (Ob-Rb) expressed in leptin-sensitive neuronal populations [194,195]. There is an assumption that the reduced expression of Ob-Rb in the brain and peripheral tissues is one of the crucial reasons for leptin resistance [196]. In rodents, age-related decline in leptin receptor expression was detected in the brain, indicating a relationship between aging and leptin resistance. For instance, it was reported that the amount of leptin receptor protein in the hypothalamus of aged obese rats was reduced by half compared with young animals [196]. A study by Fernández-Galaz et al. showed that older rats had lower levels of Ob-Rb mRNA in their hypothalamus in comparison with the young rats, but food restriction restored this parameter back to the levels seen in young rats [197]. Similar findings have been reported in another study, where Ob-Rb expression was markedly reduced in the brains of aged 5XFAD mice [198]. Guadalupe-Grau and coauthors found that leptin receptor expression was lower in vastus lateralis muscle biopsies from aged men (58 ± 8 years) in comparison with young men (24 ± 4 years) groups [199].

It is suggested that leptin receptor degradation is another mechanism of leptin resistance in obesity and aging, and it could be associated with increased activity of the matrix metalloproteinase-2 (Mmp-2) [200]. It has been found that HFD-induced obesity stimulated Mmp-2 protein activation within the hypothalamus with subsequent cleavage of the extracellular domain of the leptin receptor [200]. Mmp-2 is a member of a family of matrix metalloproteinase (MMP) involved in the resorption of extracellular matrix and other processes contributing to age-related diseases [201,202,203]. Mmp-2 activity increases with age in various tissues, including blood vessels, and perhaps this age-related change in Mmp-2 protein activity translates into age-related leptin receptor status [203]. In addition, physiologically elevated insulin levels can induce a dramatic increase in Mmp-2 activation in blood vessels [204].

## 4. Cdc42, Obesity, and Aging

Aging may be associated with both increased and decreased Cdc42 activity and expression, which varies for different cells and tissues. For instance, Cdc42 activity is significantly increased in vessels, adipose tissue, liver, blood cells, and kidney [49], while the downregulation of Cdc425 is observed only in the brain and some other organs during the aging process [49]. Notably, Cdc42 activity changes with age in metabolism-related organs expressing the leptin receptors, such as brain [205,206,207,208], adipose tissue [209,210], liver [206], and kidney [208,211]. Li et al. demonstrated that the hippocampus of old rats had reduced activity of Cdc42 compared with young animals, but 12 weeks of aerobic exercise significantly increased the activity of Cdc42 [205]. In contrast to the brain, Cdc42 activity increases with age in adipose tissue [209,210]. One reason for the increase in Cdc42 activity may be related to the change in adipocyte size, which is affected by age and obesity: average adipocyte size increases in middle and old age and then decreases over time [212]. Changes in adipocyte size are also associated with the reorganization of the actin cytoskeleton [213]. Although the study on the impact of obesity on Cdc42 activity and expression is limited, there are data demonstrating that after two weeks of a high-fat diet (HFD), C57BL6/J mice exhibited dramatic remodeling of the actin cytoskeleton and increased Cdc42 activity along with increased cell size and impaired insulin signal transduction in adipocytes [214]. Moreover, the activation of Cdc42 has been found to be increased in CD4+ T cells in obese children [215].

A mouse transcriptome study showed that obesity modulates Cdc42 expression in different mouse organs in an age-dependent manner [216]. Thus, microarray data obtained from 4-week- and 10-week-old lean and obese C57BL/6 mice and BTBR mice (mice line with ob/ob leptin-deficiency mutation) (GEO accession: 10785) demonstrated that obesity leads to a dramatic increase in Cdc42 expression in adipose tissue of C57BL/6 mice regardless of age, and a less significant upregulation of Cdc42 mRNA in BTBR mice, which was more robust in older animals. Increased expression of Cdc42 was also observed in obese livers, with the most significant changes observed in the obese C57BL/6 mice group at ten weeks of age, whereas in mice of the same line at four weeks of age, Cdc42 levels were unchanged in obesity. In the BTBR mice groups, the induction of Cdc42 in the liver during obesity was independent of age but was less pronounced than in C57BL/6 mice. In the pancreas of C57BL/6 mice, obesity caused a decrease in Cdc42 expression, especially in four-week-old animals, whereas older animals showed a slight reduction. Similar dynamics were observed in BTBR mice, where age also leveled out the decrease in Cdc42 expression. In the hypothalamus of mice, obesity and age did not lead to changes in Cdc42 expression. This aligns with recent findings indicating altered Cdc42 activity in the hypothalamus of aged rats, while the overall expression level of Cdc42 remained unchanged compared with their younger counterparts [205]. Thus, it is evident that obesity mostly has an increased impact on Cdc42’s ability to operate in a variety of tissues.

Cdc42 is a vital protein in angiogenesis and vasculogenesis as well, and its dysfunction can potentially lead to vascular dysfunction [217]. Mammoto et al.’s study revealed that Cdc42 activity in endotheliocytes from adipose tissue in individuals over 50 significantly increased compared with younger individuals [218]. The study shows that increased Cdc42 activity leads to an age-related increase in endotheliocyte size and senescence, decreased cell proliferation, and angiogenesis through aberrant Cdc42 -YAP1 signaling [218]. Moreover, a study by Ito et al. demonstrated that Cdc42 mediates p53-induced vascular inflammation in vivo, increases the expression of proinflammatory molecules in endothelial cells by activating the NF-κB pathway, and plays a crucial role in endothelial Cdc42 in chronic inflammation and the development of atherosclerosis [219]. Besides its impact on endothelial cells, Cdc42 regulates crucial events in adventitial progenitor cells and vascular smooth muscle cells [220,221,222]. Cdc42 controls the migration of Sca-1+ adventitial progenitor cells, leading to the accumulation of VSMC during vascular wall remodeling [220,222]. The activation of Cdc42 by protein kinase C delta type initiates the production of type I collagen from vascular smooth muscle cells [221]. Aging increases Cdc42 signaling in VSMC [223], potentially increasing collagen I secretion and leading to vascular fibrosis and atherosclerosis complications [224]. Moreover, overexpression of Cdc42 may increase arterial stiffness due to enhanced osteogenic differentiation of VSMC and subsequent vascular calcification [225]. Even though the precise relationship between Cdc42, insulin, and leptin in blood vessels is unknown, it is reasonable to assume that Cdc42 activation is linked to vascular remodeling caused by age-related hyperinsulinemia and hyperleptinemia. Aging exacerbates obesity-induced vascular pathology, whereas activation of Cdc42 regulates vascular function positively in young organisms [226,227].

Although gender changes in Cdc42 expression and activity are not well understood, limited research suggests that sex hormone influences are closely related to Cdc42 activity [228,229,230,231]. For instance, it has been shown that estrogens inhibit the activity of Cdc42 [230], and androgens, on the contrary, activate Cdc42 [231]. Additionally, Cdc42 has been demonstrated to inhibit the transcriptional activity of estrogen receptor alpha [228]. Clinical studies also partially support the connection between Cdc42 and estrogens: a recent study found increased expression of the Cdc42 gene in saliva associated with menopausal status, with the most significant increase observed in women over 45 years of age [229]. These observations may suggest that age-related declines in estrogen and androgen levels may influence Cdc42 regulatory pathways with subsequent changes in cellular processes and energy homeostasis.

Table 1 comprehensively outlines the multifaceted impact of age-induced changes in Cdc42 activity on the development and progression of obesity.

## 5. Crosstalk between Cdc42 and Adipoinsular Axis

As mentioned earlier, Cdc42 is a Rho GTPase protein that plays a vital role in the regulation of many cellular functions [50] and metabolic processes [238]. Several studies have identified a novel role for Cdc42 in maintaining glucose metabolism and controlling blood glucose levels through the regulation of cellular processes in metabolically active tissues such as skeletal muscle and adipose tissue, as well as the pancreas [48]. Cdc42 activates the second phase of glucose-induced insulin secretion in pancreatic islets by regulating granule fusion, exocytosis, and actin cytoskeleton rearrangement [239,240]. In addition, it is hypothesized that Cdc42 may influence pancreatic β-cell proliferation through the activation of effectors like PAK1 and CyclinD1 [48]. Furthermore, in 3T3-L1 adipocytes, Cdc42 activation mediates insulin-stimulated GLUT4 translocation and glucose transport in a PI3-kinase-dependent manner [241]. In turn, insulin treatment can enhance the Cdc42 activity and the association of Cdc42 with activated PI3-kinase (PI3K) in these cells [241]. It is worth mentioning here that PI3K plays an important role in the body’s energy balance in a variety of organs [242], and the PI3K/AKT pathway is critical for energy metabolism in insulin-sensitive tissues [242].

Veluthakal et al. demonstrated that despite the same expression level of Cdc42 in islets of people with and without type 2 diabetes (T2DM), glucose barely activated Cdc42 in islets of people with T2DM [243]. It was established that patients with T2DM have an 80% loss of expression of the critical effector of Cdc42, PAK-1, involved in the regulation of insulin secretion in comparison with healthy people [244]. In addition, a comprehensive bioinformatic analysis of 981 genes revealed that Cdc42 could potentially be used as a candidate gene target for diagnosing and treating this disease [245]. The latest research on the effect of Cdc42 deletion in pancreatic β-cells and hypothalamus showed that Rip-CDC42cKO mice exhibited glucose intolerance, a significant decrease in glucose-induced insulin secretion in isolated islets, and decreased insulin sensitivity in peripheral tissues [141]. The possible role of Cdc42 in the development of peripheral insulin resistance is evidenced by studies that have shown altered activity of Cdc42-related insulin signal transduction factors such as Cdc42 interacting protein-4 (CIP4) and G protein-coupled receptor kinase 2 (GRK2) in adipose tissue [211,246] and C9orf72 in the liver [247].

Change in lipid metabolism caused by obesity and aging is a condition known as atherogenic dyslipidemia. This condition is characterized by increased levels of triglycerides (TG) and/or low-density lipoprotein (LDL) and decreased levels of high-density lipoprotein (HDL) [248,249,250]. Epidemiological studies consistently show that atherogenic dyslipidemia is a significant risk factor for the development of atherosclerosis and metabolic disorders [101,102,251,252,253]. In turn, disruption of insulin signaling may be one of the causes of atherogenic dyslipidemia in aging and obesity [254]. Hyperinsulinemia stimulates the production of VLDL triglycerides [255,256], and elevated insulin secretion raises the concentration of low-density lipoprotein cholesterol [257].

Insulin resistance, which is inversely correlated with HDL and positively correlated with TG and LDL, is another critical factor that significantly contributes to the development of atherogenic dyslipidemia. [252,256]. Insulin resistance is closely linked to obesity, forming a dynamic interaction that has long intrigued researchers, and the intricate cause-and-effect relationship between insulin and insulin resistance remains a subject of ongoing debate [258]. However, recent findings suggest that hyperinsulinemia may precede the onset of insulin resistance [102,259]. Studies using mutant mice with impaired insulin clearance revealed impaired insulin clearance and hyperinsulinemia at 2 months, followed by hepatic insulin resistance at 6–7 months. This was subsequently accompanied by visceral obesity, hyperphagia, hyperleptinemia, and hypothalamic leptin resistance [260].

An age-dependent [248] or obesity-induced [261,262] increase in free fatty acid (FFA) levels is considered a likely mechanism causing changes in lipid metabolism through impaired insulin signaling. It has been demonstrated that sharply increasing FFA can reduce insulin action [263] and cause adipose tissue to express and secrete proinflammatory cytokines, which, in turn, reduce insulin sensitivity [264]. Einstein et al. demonstrated that the inflammatory response to nutrient excess and susceptibility to FFA-induced insulin resistance are exacerbated by aging [265]. One of the major signaling pathways involved in the development of FFA-induced liver insulin resistance is the c-Jun N-terminal kinase (JNK) pathway, which contributes to the disruption of insulin signaling and promotes cell damage (lipoapoptosis) [266,267]. In turn, Cdc42 is involved in the regulation of liver lipid status as a major contributor to the saturated fatty acid-stimulated JNK pathway in hepatocytes, which promotes lipoapoptosis and NAFLD [268]. In addition, Cdc42 activation leads to increased fluid-phase pinocytosis of LDL by macrophages [234,235,236], causing them to absorb excess lipids and turn into foam cells [269], thereby enhancing in vivo atherosclerosis [236]. Therefore, age-related activation of Cdc42 in the liver and macrophages may contribute to dyslipidemia and atherosclerosis.

On the other hand, hyperleptinemia is a significant contributor to obesity and the disruption of energy homeostasis [270,271,272]. Under physiological conditions, leptin enhances insulin sensitivity by increasing fatty acid oxidation and glucose uptake through AMPK and PPAR-alpha activation [273]. Hyperleptinemia leads to an increase in insulin resistance and disruption of glucose metabolism [270,271,272]. A partial decrease in plasma leptin levels in obese patients can restore hypothalamic leptin sensitivity, reduce weight gain, and improve insulin sensitivity [271]. Upregulation of Cdc42 in obesity contributes to the development of insulin resistance through increased leptin production by hypertrophied adipocytes. Moreover, the possible role of Cdc42 in the development of peripheral insulin resistance is evidenced by studies that have shown altered activity of Cdc42-related insulin signal transduction factors such as Cdc42 interacting protein-4 (CIP4) and G protein-coupled receptor kinase 2 (GRK2) in adipose tissue [211,246] and C9orf72 in the liver [247].

Considering the close relationships between obesity, leptin resistance, insulin resistance, and aging, it is conceivable that Cdc42 dysfunction may be associated with leptin resistance [270,274]. Leptin has many different influences on Cdc42 activity depending on the type of cells it acts upon. Several studies described interactions with leptin and Cdc42 during neurogenesis in the hippocampus, in which leptin exposure activated Cdc42 in hippocampal neurons [275,276,277]. It has been reported that exposure of hippocampal neurons to leptin triggers a CaMKK/CaMKI signaling cascade that leads to phosphorylation and activation of β1Pix, a Cdc42/Rac guanine nucleotide exchange factor (GEF) [275]. It was also found that the leptin receptor and β-PIX form a complex, the amount of which temporarily increases upon activation of the leptin receptor during GABAergic synaptogenesis in hippocampal neurons [276]. In another study, leptin was shown to suppress the expression of p250GAP, a negative regulator of Rac, RhoA, and Cdc42, and this suppression is mandatory for enhancing synaptogenesis in hippocampal neurons [277].

The interaction between leptin and Cdc42 occurs not only in the brain but also in peripheral organs. Leptin exposure activates Cdc42 in mouse Sca-1+ vascular progenitor cells [222] and human brain microvascular pericytes [227] and causes enhanced angiogenesis and cell migration. The identification of mRNAs for differentially expressed genes after leptin treatment of *ob/ob* mice showed a 1.5-fold increase in Cdc42 expression in the liver [278]. In contrast, in rat cardiomyocytes, leptin did not affect Cdc42 activity [279]. In some studies, leptin induced PI3K-dependent reorganization of the actin cytoskeleton, leading to the depolymerization of F-actin in pancreatic β-cells of mice and rats [280,281]. Ning et al. discovered that leptin-induced F-actin depolymerization is linked to PTEN inhibition [281], which can activate Cdc42, causing actin cytoskeleton reorganization [136]. If considering the PI3K signaling in the brain, it is shown that it has a central role in the regulation of satiety by leptin [282]. In this regard, inhibiting PI3K by LY294003 abrogated the reduction in food intake stimulation by leptin [283]. In the ARC, leptin activates PI3K in the POMC neurons while indirectly inhibiting PI3K in the NPY/AgRP neurons [284], leading to suppression of food intake. In vivo, studies with neuron-specific PI3K ablation have pointed to a role of PI3K in leptin-mediated feeding behavior and body weight regulation. Mice with POMC-specific depletion of PI3K regulatory subunits p85α and p85β have normal food intake and body weight but fail to suppress food intake upon acute leptin administration [282]. Considering the fact that PI3K binds to Cdc42 via the N terminal region of the p85α regulatory subunit [241], it is suggested that both proteins may affect energy metabolism via a common signaling pathway.

In turn, Cdc42 may influence leptin secretion by mediating insulin action. Numerous studies have found insulin-induced long-term leptin secretion by fat cells through a transcriptional or post-transcriptional mechanism [35,285,286,287]. Zeigerer et al. demonstrated that treatment of 3T3-L1 adipocytes with insulin caused a two-fold increase in leptin secretion and could be blocked by brefeldin A [287]. Since mammalian Cdc42 is a Brefeldin A-sensitive component of the Golgi apparatus, it can be assumed that Cdc42 is involved in leptin secretion [288].

As indicated in the previous section, one of the pivotal regulators of leptin signaling is metalloproteinase MMP-2, the activity of which leads to cleavage of the extracellular domain of the leptin receptor in the hypothalamus [200]. Cdc42 can regulate VEGF-dependent MMP-2 activation in endothelial cells, and overexpression of Cdc42 enhances MMP-2 activity and capillary sprouting [289,290]. Since increased Cdc42 and MMP-2 activity are observed in obesity and aging, it is conceivable that increased Cdc42 activity may lead to the degradation of the leptin receptor and affect leptin transport to the brain.

**Figure 1:**
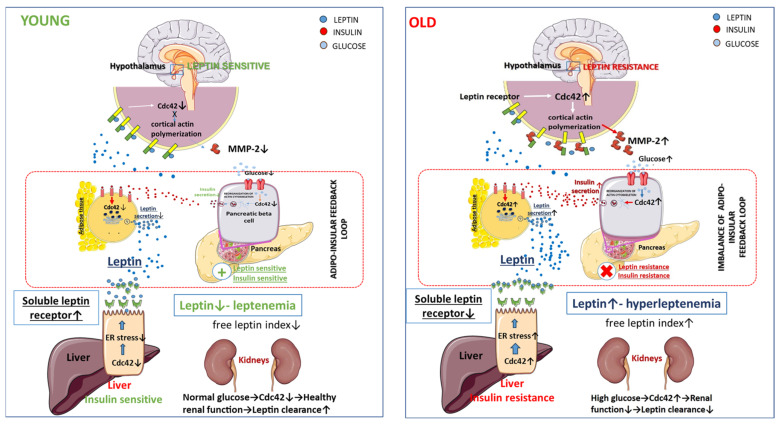
Role of Cdc42 in the imbalance of adipoinsular axis.

As discussed in the previous chapter, ER stress plays a critical role in the release of the soluble form of the leptin receptor by the liver and the development of leptin resistance [189]. Obesity can lead to the development of ER stress through the suppression of hepatic autophagy [291]. N-WASP, expressed in hepatocytes, is activated by Cdc42, leading to actin polymerization and inhibiting autophagy. A recent study showed that increased Cdc42 activity in hepatocytes associated with insulin resistance leads to the suppression of lipophagy, a type of autophagy [247]. Several lines of evidence suggest that Cdc42 levels in the liver increase with aging [206,216,232]. Therefore, it can be assumed that increased age-related activity of Cdc42 in the liver may enhance leptin resistance through a decrease in soluble forms of the leptin receptor. Moreover, Cdc42 may have indirect effects on the development of leptin resistance through the regulation of leptin clearance. The main organ involved in leptin excretion is the kidneys. It excretes up to 80% of leptin, and renal dysfunction may actively contribute to hyperleptinemia [292]. Therefore, an indirect effect of Cdc42 on leptin levels is possible through renal dysfunction with Cdc42 activation in podocytes and renal vascular cells caused by hyperglycemia [225,293,294,295]. Table 2 below summarizes the relationship between Cdc42 and the adipoinsular axis. We suggest that Cdc42 is an important element of the adipoinsular axis and age-related dysregulation of Cdc42 may exacerbate metabolic disturbances and lead to age-related obesity (Figure 1).

## 6. The Potential Therapeutic Implications of Cdc42 and Adipoinsular Axis to Mitigate Obesity

It is noteworthy to mention that some drugs used to sensitize tissues to leptin and affect obesity can modulate Cdc42 activity. Thus, the antidiabetic drug metformin, which has been reported to be able to reduce leptin and insulin levels and increase the expression of the leptin receptor gene (Ob-Rb) in the arcuate nucleus [313], has an inhibitory effect on Cdc42 [314]. Moreover, curcumin, a lipophilic polyphenol derived from the spice turmeric, which reduces weight, leptin, and leptin levels in patients with metabolic syndrome and related disorders [315,316], can inhibit Cdc42 activity [317].

Several clinical studies have shown that the polyphenolic compound with pleiotropic activity, resveratrol (trans-3,5,4′-trihydroxystilbene), can be used as a therapeutic strategy in the control of obesity [318]. The exact mechanisms of resveratrol’s effects on adipose tissue and glucose metabolism are not known, but it is interesting that resveratrol suppresses glucose-induced vascular smooth muscle cell migration via inhibition of Cdc42 [319]. However, these agents affect a wide range of signaling pathways, and it is difficult to assess the contribution of Cdc42 inhibition to their metabolic effects.

Long-chain omega-3 polyunsaturated fatty acids (*n*-3 PUFAs) have anti-inflammatory and hypotriglyceridemic properties, so the use of a diet based on them is being considered for the treatment and prevention of obesity and related diseases [320]. It has been shown that a diet rich in *n*-3 PUFAs improves insulin sensitivity and may prevent the development of insulin resistance in response to high-fat feeding and modulates the expression and secretion of adipocytokines [321,322]. Moreover, a diet based on *n*-3 PUFAs has a major impact on influencing leptin in the context of inflammation in obesity [323]. In addition, a 12-week open-label intervention study showed that consumption of 2.7 g/day of omega-3 polyunsaturated fatty acids led to a decrease in the level of Cdc42 mRNA in the blood of men [324]. In vitro and in vivo studies revealed that diets enriched with omega-3 polyunsaturated fatty acids suppressed Cdc42 activity in mice colonocytes [325].

These data suggest that nutritional interference of Cdc42 is possible, but the effects of different nutrients on Cdc42 regulatory dynamics are not fully understood and require further study. Furthermore, dysregulation of Cdc42, triggered by dietary factors or aging, may contribute significantly to the development of insulin and leptin resistance, highlighting its central importance in metabolic health. Although we have not found any studies on the effect of selective Cdc42 inhibitors on obesity, it has been shown that systemic administration of a selective small molecule, the Cdc42 inhibitor CASIN, can significantly reduce inflammatory processes in the body and increase life expectancy [237]. Therefore, it could be of great interest to evaluate the effects of Cdc42 inhibitors and activators on leptin resistance and obesity.

## 7. Conclusions

Obesity in middle age has profound implications, hastening the onset of chronic diseases and disability. Central to this process is the disruption of the adipoinsular axis due to aging, a crucial factor contributing to obesity. Although leptin and insulin are the key players that regulate adipose tissue homeostasis and energy metabolism, the complex interplay between these two hormones is still poorly understood, highlighting the need for the identification of new molecular targets that interconnect insulin and leptin signaling networks. In this regard, our study highlights the role of Rho GTPase protein Cdc42 as an important component of this complex network. Dysregulation of Cdc42, whether induced by external or internal factors, appears to be an important contributor to the development of leptin and insulin resistance. This dysregulation is prominent in a variety of metabolically active organs such as the brain, pancreas, adipose tissue, liver, and kidneys. The effects of Cdc42 have been confirmed in pancreatic β-cells and the hypothalamus, revealing striking expression patterns in glucose tolerance, insulin secretion, and insulin sensitivity. The association between increased Cdc42 activity and inhibition of autophagy in hepatocytes sheds light on potential mechanisms underlying obesity-related insulin resistance. In turn, Cdc42 may influence leptin secretion by mediating insulin action and Cdc42 dysfunction may be associated with leptin resistance. These discoveries open up promising therapeutic avenues for managing obesity and associated disorders. Potential interventions targeting Cdc42 activity hold promise for reducing obesity and enhancing leptin sensitivity. These novel approaches could revolutionize obesity treatments. However, owing to the tissue-specific nature of Cdc42 activity, it is imperative to tailor interventions aiming at different tissues and employ targeted delivery methods to minimize systemic side effects. Further in-depth studies are essential to thoroughly assess the effectiveness and safety of selective Cdc42 inhibitors.

## Figures and Tables

**Table 1:** Age-related Cdc42 function and possible effects on obesity.

Type	Direction	Possible Impact on Obesity	References
Endotheliocytes	Upregulation	Atherosclerosis↑ and vascular remodeling ↑ Cell senescence↑ Secretion of proinflammatory cytokines↑ Activation of MMP2 ↑ (Leptin receptor signaling ↓)	[218,219,220,222]
Vascular smooth muscle cells	Upregulation	Calcification of kidney vessels ↑	[223,225]
Hepatocytes	Upregulation	Impairment of liver lipid homeostasis ↑ ER stress ↑ (Soluble leptin receptor ↓)	[206,216,232]
Adipose-derived mesenchymal stem cells/adipocytes	Upregulation	Adipogenesis ↓ Cell hypertrophy ↑ Leptin production ↑	[209,210]
Monocytes/macrophages	Upregulation	Chronic inflammation ↓ Pinocytosis of LDL by macrophages ↑ (transformation to foam cells ↑) Secretion of proinflammatory cytokines ↑	[233,234,235,236,237]

**Table 2:** Adipoinsular axis and Cdc42.

Type of Regulation	Adipoinsular Axis	Insulin Receptors (IRs)	Leptin Receptors	Cdc42
Insulin ↑	Leptin expression and secretion in human adipocytes ↑ [296] Leptin in human WAT ↑ [297] Leptin in human SAT ↑ [298] Leptin in rat VAT [299]	IRs in mouse hippocampal microvessels ↓ [300] IRs in human adipocytes ↓ [301] PTP1B in rat artery ↓ [302] PTP1B in mouse liver ↑ [303]	PTP1B in rat artery ↓ [302] PTP1B in mouse liver ↑ [303] MMP-2↑ in rat blood vessels [204]	Cdc42 activity in 3T3-L1 adipocytes ↑ [241] Cdc42 activity in rat hippocampal neurons ↑ [304]
Leptin ↑	Serum insulin in ob/ob mice ↓ [296] Insulin secretion and insulin mRNA levels in isolated pancreatic islets of rat ↓ [305] Insulin secretion and insulin mRNA levels in human pancreatic Islets ↓ [306] Insulin secretion in insulinoma cells, rat and human islets and, in vivo, in mice ↓ [307]	PTP1B in the hypothalamus of DIO mice ↑ [272] PTP1B in the liver of ob/ob mice ↑ [308] PTP1B in the muscle and fat of ob/ob mice unchanged [308] PTP1B in human hepatoma cells (HepG2) ↑ [308]	Hypothalamic Ob-Rb in rats ↓ [309,310] Ob-Re human embryonic kidney (HEK) 293 cells [189] MMP-2↑ 3T3-L1 adipocytes↑ [311]	β1Pix in rat hippocampal neurons ↑ [275,276] p250GAP in hippocampal neurons of db/db mice ↓ [277] PTEN in mouse beta cells of the pancreas ↓ [281] Cdc42 activity in mouse Sca-1+ vascular progenitor cells ↑ [222] and human brain microvascular pericytes ↑ [227] Cdc42 expression↑ in ob/ob mice liver [278] Cdc42 activity in rat cardiomyocytes unchanged [279]
Cdc42 ↑	Second-phase insulin secretion in human islet culture and MIN6 cell culture ↑ [240,312] Leptin secretion in 3T3-L1 adipocytes ↑ [287]	Insulin-stimulated Glut4 translocation in 3T3L1 adipocytes ↑ [241,246]	ER stress in mouse hepatocytes ↑ [247] → Ob-Re ↓ MMP-2 ↑ in rat endotheliocytes [289,290]	

WAT—White adipose tissue, SAT—subcutaneous adipose tissue, VAT—visceral adipose tissue, IRs—insulin receptors, Ob-Rb—the long form of the leptin receptor, Ob-Re—soluble receptor of leptin, ob/ob mice—mice characterized by a mutation of the obese (ob) gene encoding leptin, DIO mice—diet-induced obese mice, PTP1B—protein tyrosine phosphatase 1B, β1Pix—guanine nucleotide exchange factor (GEF) for Cdc42, MMP-2—matrix metalloproteinase-2, Glut4—glucose transporter type 4, and ER stress—endoplasmic reticulum stress.
